# Impact of surface-guided prepositioning and respiratory coaching on the target localization accuracy in lung stereotactic body radiation therapy

**DOI:** 10.1007/s12194-026-01008-2

**Published:** 2026-02-23

**Authors:** Kazuki Onishi, Naoki Hayashi, Tatsunori Saito, Yuta Muraki, Shinya Neri, Masashi Nozue

**Affiliations:** 1 Department of Radiology, Seirei Hamamatsu General Hospital, Hamamatsu, Shizuoka 430-8558 Japan; 2 Division of Medical Physics, School of Medical Sciences, Fujita Health University, 1-98, Dengakugakubo, Kutsukake-cho, Toyoake, Aichi 470-1192 Japan; 3 Department of Radiation Oncology, Seirei Hamamatsu General Hospital, Hamamatsu, Shizuoka 430-8558 Japan

**Keywords:** Correlation, Internal target, Surface guidance, Stereotactic body radiotherapy

## Abstract

This study assessed the effect of surface-guided radiation therapy (SGRT)-based prepositioning and respiratory coaching on target localization accuracy in lung stereotactic body radiation therapy (SBRT) using deep inspiration breath-holding. Thirty-five patients treated with lung SBRT (September 2022 to March 2025) were classified into three groups: Group A, VOXELAN prepositioning with reproducible respiratory control (≥ 60% setup criteria satisfied); Group B, VOXELAN prepositioning without reproducible control; and Group C, no VOXELAN prepositioning. Cone-beam computed tomography (CBCT) after prepositioning was used to retrospectively assess target localization. The concordance between the VOXELAN setup criteria and CBCT errors (> 5 mm) was analyzed using Fisher’s exact test. Positional deviations and rotations were compared among groups using analysis of variance and post-hoc tests. Satisfying the VOXELAN setup criteria significantly correlated with CBCT localization within 5 mm (*p* = 0.0027). Vertical errors were smaller in Groups A and B than in Group C (*p* < 0.01), and lateral errors were smaller in Groups A and B than in Group C (*p* = 0.01 and *p* < 0.01, respectively). Rotational errors were within ± 1° in all groups, with a significant difference between Groups A and C (*p* < 0.02). Longitudinal errors were not significantly different between the groups. SGRT-based prepositioning with respiratory coaching improved setup reproducibility and correlation with the internal target position, particularly in the vertical, lateral, and rotational axes. Longitudinal accuracy remained limited, suggesting caution in margin reduction.

## Introduction

Surface-guided radiation therapy (SGRT) has emerged as a pivotal technique in modern radiation oncology, utilizing optical surface imaging to enhance patient setup accuracy and monitor intra-fractional motion [1]. This method offers a non-invasive alternative to traditional imaging modalities, aiming to improve treatment precision and patient safety [2, 3]. Notably, SGRT has been reported to improve setup accuracy in lung stereotactic body radiotherapy (SBRT) and enable the planning target volume (PTV) margin reduction [4]. Its advantages include real-time monitoring of radiation delivery, non-ionizing imaging, enhanced patient comfort, and the application of the breath-hold techniques [5]. Nonetheless, the surface-to-volume correlation in patients, dependence on surface visibility, initial setup, calibration [6], and cost and resource allocation are well-known disadvantages.

VOXELAN (ERD Corporation, Okayama, Japan) is an SGRT device that acquires surface signals and performs prepositioning before radiation delivery, as well as managing patient respiration [7–9]. Compared to other SGRT devices, VOXELAN can display the body surface outline in real time using the light-section method by projecting two red lasers onto the patient simultaneously [10]. This enables real-time monitoring of patient position and respiratory-induced chest or abdominal wall motion without additional equipment. Therefore, VOXELAN was installed in the simulation computed tomography (CT) room to acquire chest and abdominal wall motion data with respiratory coaching prior to CT scanning, and these data were used for prepositioning in the treatment room. Respiratory motion control methods such as deep inspiration breath-hold (DIBH) and abdominal compression have also been shown to reduce setup variability during lung SBRT [11]. Therefore, we installed VOXELAN in the simulation CT room to obtain chest/abdominal wall motion data with respiratory motion coaching prior to simulation CT scanning to implement the data for prepositioning in the treatment room.

SBRT to the lung is sensitive to patient motion because of the high fractional dose and low number of fractions delivered [12]. For a small-fraction dose, the effect of missing the target was smaller than that for a high-fraction dose used in SBRT [13]. In this case, the effects include both a potential reduction in the total dose to the target and damage to healthy tissue [14]. Thus, the dosimetric consequences are greater for SBRT if the target is affected by motion due to respiration. Therefore, respiratory management is extremely important in SBRT as well as in the position of the patient during radiation delivery [15, 16]. Furthermore, accurate setup and respiratory control facilitate individualized PTV margin strategies according to the tumor location and motion characteristics [17].

At our hospital, we use DIBH radiation therapy with tumor matching using cone-beam computed tomography (CBCT) for localization after prepositioning with VOXELAN. During radiation delivery, the position of the patients and respiratory signals are constantly monitored using VOXELAN. However, the correlation between the position of the body surface detected by VOXELAN and that of the target is unclear, as is the extent to which the SGRT device contributes to improving the accuracy of the irradiation position. Additionally, the effect of coaching on respiratory management and setup accuracy needs to be evaluated.

This study aimed to evaluate the impact of SGRT-based prepositioning and respiratory coaching on target localization accuracy in lung SBRT under DIBH conditions.

## Materials and methods

A. ***Definition of prepositioning***

In this study, prepositioning refers to the initial patient setup process performed immediately before cone-beam computed tomography (CBCT) acquisition, in which the patient’s body surface captured by the VOXELAN system is aligned with the reference surface obtained during simulation. The purpose of this step is to minimize any residual setup deviation before image-guided tumor matching. The setup criterion for the prepositioning procedure was defined as translational and rotational errors within 3 mm and 2°, respectively, as displayed by VOXELAN under respiratory coaching during simulation CT. This criteria was defined at the time of starting this research. During prepositioning, these offsets are adjusted in real time by radiological technologists until the surface error meets the predefined criteria (≤ 3 mm and ≤ 2°). This procedure is distinct from respiratory training, which is conducted prior to simulation CT to help patients learn reproducible breath-hold or shallow-breathing patterns.

B. ***Patient distribution***

**Fig. 1:**
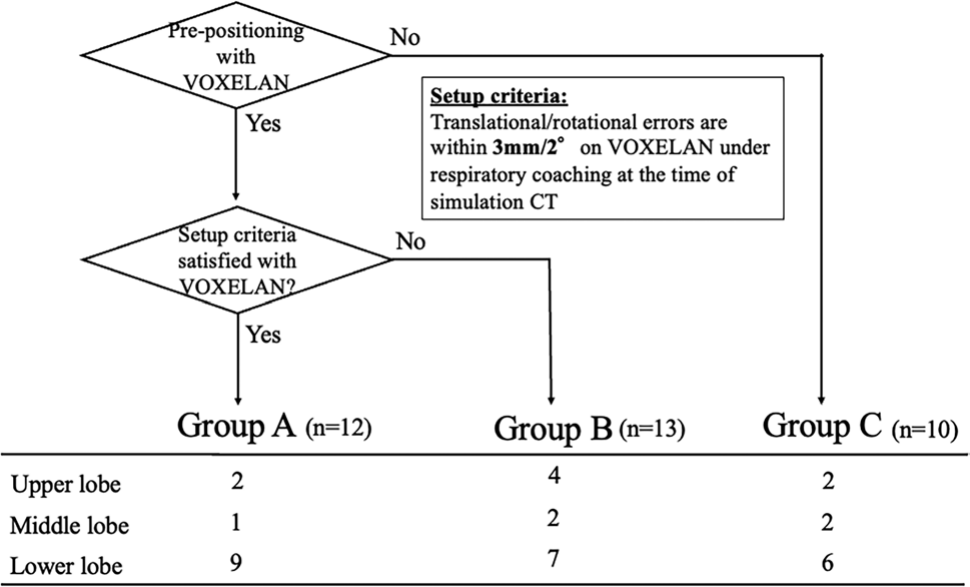
Classification of patients analyzed in this study. CT, computed tomography

All lung SBRTs at our hospital were performed using TrueBeam linear accelerator (Siemens-Varian Corporation, Germany) with the DIBH technique. To evaluate the effectiveness of the patient setup and respiratory management using VOXELAN, we investigated 35 patients who underwent lung SBRT at our hospital between September 2022 and March 2025. The detailed conditions of the 35 cases are shown in Fig. 1. In this study, the prepositioning and respiratory-coaching criteria were established in advance to assess their influence on target localization accuracy. Based on our preliminary institutional evaluation, achieving ≥ 60% compliance with the setup criteria (translational/rotational errors within 3 mm/2° on VOXELAN) was considered clinically feasible without workflow disruption. Group A comprised patients for whom prepositioning with VOXELAN was performed and whose respiratory control during the treatment-planning CT could be successfully reproduced at treatment. This group satisfied ≥ 60% of the setup criteria using VOXELAN. Group B included patients for whom prepositioning was performed using VOXELAN; however, respiratory control at the time of treatment planning CT could not be reproduced. Group C included patients in whom prepositioning was not performed using VOXELAN. Groups A, B, and C comprised 12, 13, and 10 patients in 66, 79, and 70 fractions, respectively. This study was approved by the Institutional Review Board (IRB) of our hospital, and all patients provided consent to participate in the study (IRB approval number SH-4397).

C. ***Setup flow of SBRT***

**Fig. 2:**
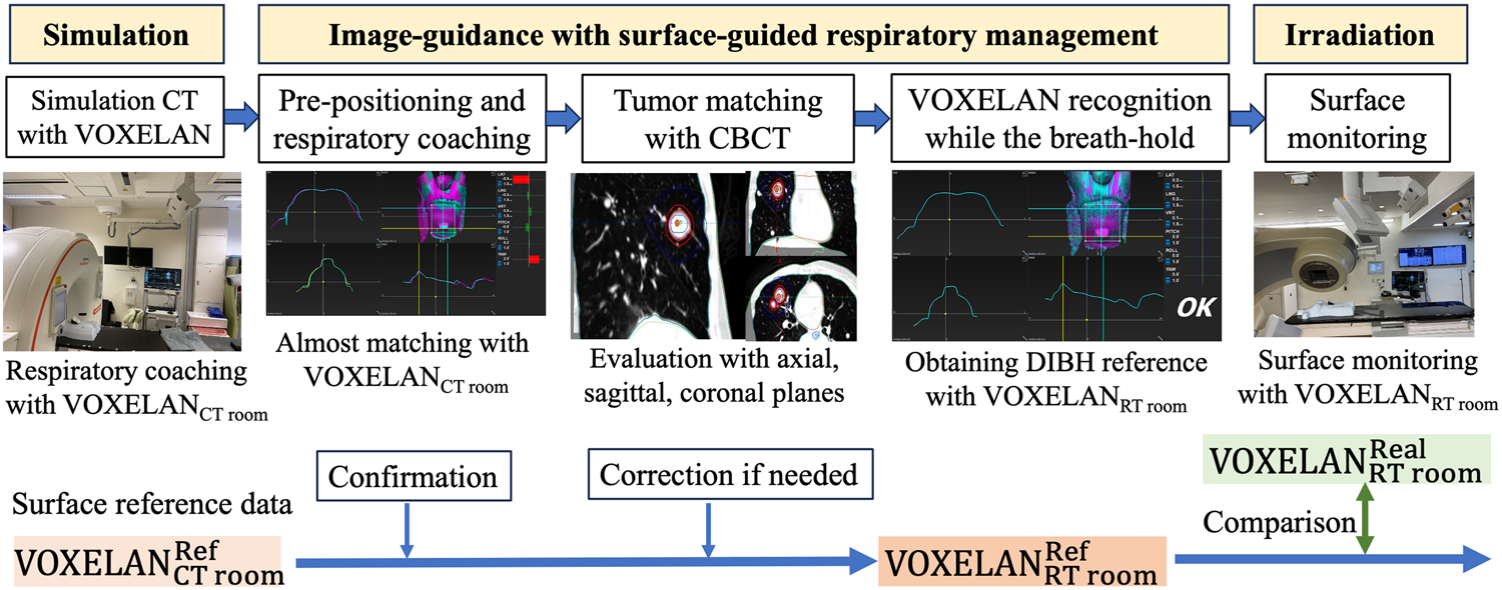
Setup and monitoring workflow with VOXELAN. *CBCT* cone-beam computed tomography, *CT* computed tomography, *DIBH* deep inspiration breath-hold, *Ref* reference, *RT* radiotherapy

Figure 2 displays the setup flow of the SBRT at our hospital. First, we performed simulated CT under respiratory management (coaching) to accurately reproduce breath-holding. The slice thickness of the simulated CT was 1 mm during the breath-holding phase for all patients. In this phase, surface reference data with VOXELAN in a simulation CT room ($\:{\mathrm{V}\mathrm{O}\mathrm{X}\mathrm{E}\mathrm{L}\mathrm{A}\mathrm{N}}_{\mathrm{C}\mathrm{T}\:\mathrm{r}\mathrm{o}\mathrm{o}\mathrm{m}}^{\mathrm{R}\mathrm{e}\mathrm{f}}$) were created for all patients. When performing respiratory coaching in the simulation CT room, we confirm that the body surface profile is repeatedly reproduced on the VOXELAN screen that installed in the CT room while the patient performs multiple breath-holds. The radiological technologist provides feedback to the patient regarding tidal volume by referring to this screen.

During the radiation delivery phase in the treatment room, prepositioning using VOXELAN was performed before acquiring the CBCT images. Here, $\:{\mathrm{V}\mathrm{O}\mathrm{X}\mathrm{E}\mathrm{L}\mathrm{A}\mathrm{N}}_{\mathrm{C}\mathrm{T}\:\mathrm{r}\mathrm{o}\mathrm{o}\mathrm{m}}^{\mathrm{R}\mathrm{e}\mathrm{f}}$ was compared to the surface data obtained by VOXELAN in the treatment room. If surface matching was acceptable within the criteria, the data were converted to surface reference data in the treatment room ($\:{\mathrm{V}\mathrm{O}\mathrm{X}\mathrm{E}\mathrm{L}\mathrm{A}\mathrm{N}}_{\mathrm{R}\mathrm{T}\:\mathrm{r}\mathrm{o}\mathrm{o}\mathrm{m}}^{\mathrm{R}\mathrm{e}\mathrm{f}}$) without correction. If the surface data were unacceptable, $\:{\mathrm{V}\mathrm{O}\mathrm{X}\mathrm{E}\mathrm{L}\mathrm{A}\mathrm{N}}_{\mathrm{C}\mathrm{T}\:\mathrm{r}\mathrm{o}\mathrm{o}\mathrm{m}}^{\mathrm{R}\mathrm{e}\mathrm{f}}$ was corrected following the tumor-matching position using CBCT to create a new $\:{\mathrm{V}\mathrm{O}\mathrm{X}\mathrm{E}\mathrm{L}\mathrm{A}\mathrm{N}}_{\mathrm{R}\mathrm{T}\:\mathrm{r}\mathrm{o}\mathrm{o}\mathrm{m}}^{\mathrm{R}\mathrm{e}\mathrm{f}}$. After confirming that the position of the patient could be reproduced during prepositioning, we used VOXELAN to monitor the patient surface ($\:{\mathrm{V}\mathrm{O}\mathrm{X}\mathrm{E}\mathrm{L}\mathrm{A}\mathrm{N}}_{\mathrm{R}\mathrm{T}\:\mathrm{r}\mathrm{o}\mathrm{o}\mathrm{m}}^{\mathrm{R}\mathrm{e}\mathrm{a}\mathrm{l}}$) to determine whether the same breathing control (breath-hold) as that during the treatment planning CT could be achieved. Patients who could achieve the same respiratory control level as that during the treatment planning CT and who satisfied the setup criteria in the deep inspiration hold state on VOXELAN were included in Group (A) Conversely, those who could not achieve the same level of control as that during the treatment planning CT or who did not satisfy the position accuracy criteria in the deep inspiration hold state comprised Group (B) The setup criterion was a translational shift error and rotational angle error of 3 mm/2°. After confirming the reproducibility of patient positioning and respiratory management during the prepositioning process, patient localization was performed using CBCT. Patient localization was conducted using tumor matching, and the tumor position was matched to the same position as that in the treatment-planning CT. Following confirmation that the position of the tumor was correct under imaging guidance, radiation was delivered.

D. ***Data analysis***

To evaluate the feasibility of the VOXELAN setup criteria (Fig. 1), we analyzed the matching between the VOXELAN setup criteria and the positional error detected using CBCT. The patients in groups A and B were included in this evaluation. The CBCT data were categorized as “OK” if the error was ≤ 5 mm and as “NG” if the error was > 5 mm in CBCT. Fisher’s exact test was applied to compare the two groups according to the VOXELAN setup criteria (OK/NG) and positional error on CBCT (OK/NG) as the statistical analysis method.

Furthermore, the reproducibility of the target position was retrospectively evaluated using CBCT images with localization for all patients. Offline Review software Version 16.1 (Siemens-Varian Corporation, Germany) was used to analyze the CBCT images. When analyzing the images using the Offline Review software, the vertebrae on the isocenter plane were set as landmarks to calculate the distance between the isocenter position set in the tumor and the vertebrae. This measurement was also performed on treatment-planning CT images, and the deviation was calculated by subtracting the results of the CBCT images from those of the treatment-planning CT images. All analyses were performed by the same radiological technologist to eliminate any observer differences. The equation for calculating the discrepancy between the actual target position and the target positions at the time of treatment-planning CT is as follows:

1 $$\:\mathrm{D}\mathrm{i}\mathrm{f}\mathrm{f}\mathrm{e}\mathrm{r}\mathrm{e}\mathrm{n}\mathrm{c}\mathrm{e}=\mathrm{a}\mathrm{c}\mathrm{t}\mathrm{u}\mathrm{a}\mathrm{l}\:{\mathrm{p}\mathrm{o}\mathrm{s}\mathrm{i}\mathrm{t}\mathrm{i}\mathrm{o}\mathrm{n}\mathrm{s}}_{\mathrm{C}\mathrm{B}\mathrm{C}\mathrm{T}}-\mathrm{t}\mathrm{a}\mathrm{r}\mathrm{g}\mathrm{e}\mathrm{t}\:{\mathrm{p}\mathrm{o}\mathrm{s}\mathrm{i}\mathrm{t}\mathrm{i}\mathrm{o}\mathrm{n}\mathrm{s}}_{\mathrm{s}\mathrm{i}\mathrm{m}\mathrm{C}\mathrm{T}}$$

Data were statistically analyzed using one-way analysis of variance and t-tests with the Bonferroni correction.

## Results

Table 1 presents a crossover table classified according to whether the VOXELAN setup criteria were met and whether there was an error > 5 mm on the CBCT image. The VOXELAN setup criteria specified that the positioning accuracy should be a translational shift error/rotational angle error of 3 mm/2° (see Fig. 1). Because Fisher’s p-value was 0.0027, there was good agreement between meeting the VOXELAN setup criteria and detecting a 5 mm error was detected by CBCT.

**Table 1:**
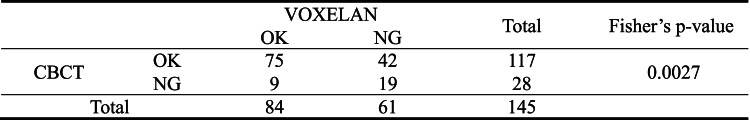
Crossover table showing classification between VOXELAN setup criteria and CBCT matching

CBCT, cone-beam computed tomography

**Fig. 3:**
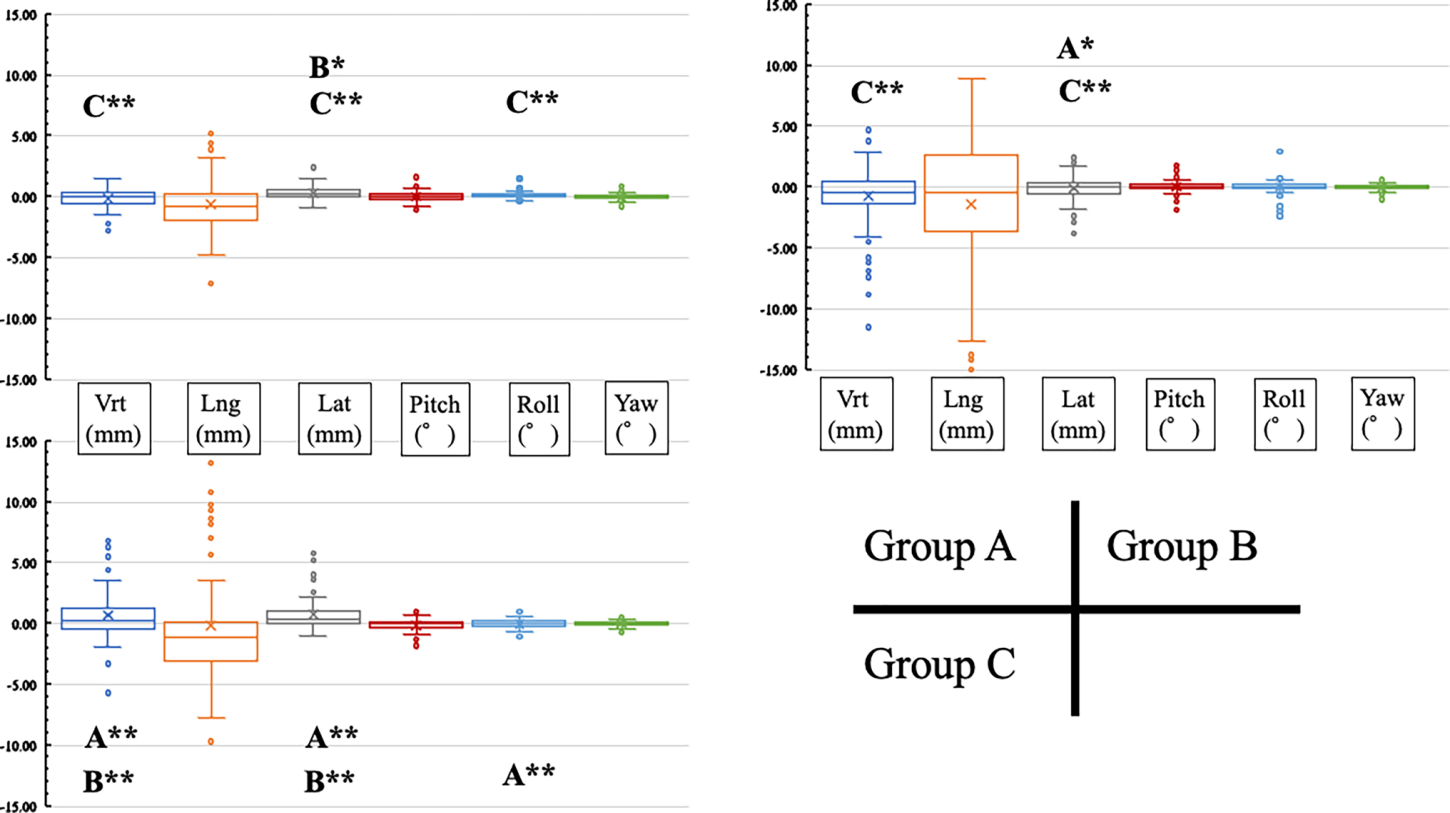
Discrepancies between the actual target position and the target positions at the time of simulation CT. The boxplot shows the interquartile range of the data in the box, with the median indicated by a horizontal line inside the box. The length of the whiskers indicates 1.5 times the interquartile range, and values that deviate from this range are indicated by points. Symbols (A-C) denote comparative groups, while * and ** indicate p-values ≤ 0.05 and ≤ 0.01, respectively. *Lat* lateral, *Lng* longitudinal, *Vrt* vertical

Figure 3 presents the discrepancies between the actual target positions and the target positions at the time of treatment planning using CT and CBCT for Groups A, B, and C. The boxplot displays the interquartile range of the data in the box, with the median indicated by a horizontal line inside the box. The length of the whiskers represents 1.5 times the interquartile range, and values that deviate from this range are indicated by points. The longitudinal error was greater in Groups A, B, and C than in other directions. The median values in the longitudinal direction for Groups A, B, and C were − 0.80 mm, −0.40 mm, and − 1.10 mm, respectively, which were slightly smaller than 0 mm, indicating a minor shift in the inferior direction compared with the simulation CT. Group A had a smaller interquartile range and whisker length than did the other groups. Nonetheless, statistical analyses (one-way analysis of variance and t-test with Bonferroni correction) did not reveal any statistically significant differences in the longitudinal direction. However, different trends were observed in the vertical and lateral directions across the three groups. In the vertical direction, statistically significant differences were observed between Groups A and C and between Groups B and C (both *p* < 0.01). The interquartile ranges differed significantly between Group C and the other groups. Although the interquartile ranges between Groups A and B did not differ significantly, the number of outliers was higher in Group B than in Group A (non-statistically significant, *p* = 0.09). In the lateral direction, there were no remarkable differences among the three groups regarding median and interquartile range; nevertheless, there were many outliers in Groups B and C. Additionally, statistical significance was observed between groups A and C and between groups B and C (*p* = 0.01, *p* < 0.01, respectively). The rotational errors were all less than ± 1°, and no major differences were observed among the three groups. Nevertheless, there was no significant difference between groups A and C (*p* < 0.02).

## Discussion

In this study, we evaluated target localization reproducibility in patients treated with SBRT using the DIBH protocol, both with and without the use of VOXELAN in prepositioning, and with and without respiratory hold coaching using VOXELAN. We divided participants into three groups, analyzed them, and found that the variation in the long direction was large in all groups. After prepositioning with VOXELAN and implementing breath-hold coaching, Group A satisfied the setup criteria and exhibited the smallest variation in the longitudinal direction; however, there was no statistically significant difference among the three groups. Although the quantitative improvement was modest, the results indicate that combining SGRT-based prepositioning with respiratory coaching can stabilize patient setup reproducibility under clinical conditions. This may be because there were cases in Group A that did not match sufficiently in the longitudinal direction. Therefore, we analyzed the results of the cases that were outliers in the longitudinal boxplot in detail. Among these, cases G and H were outliers, exhibiting lower internal target matching than did the other cases. Thus, cases G and H were investigated in detail.

**Fig. 4:**
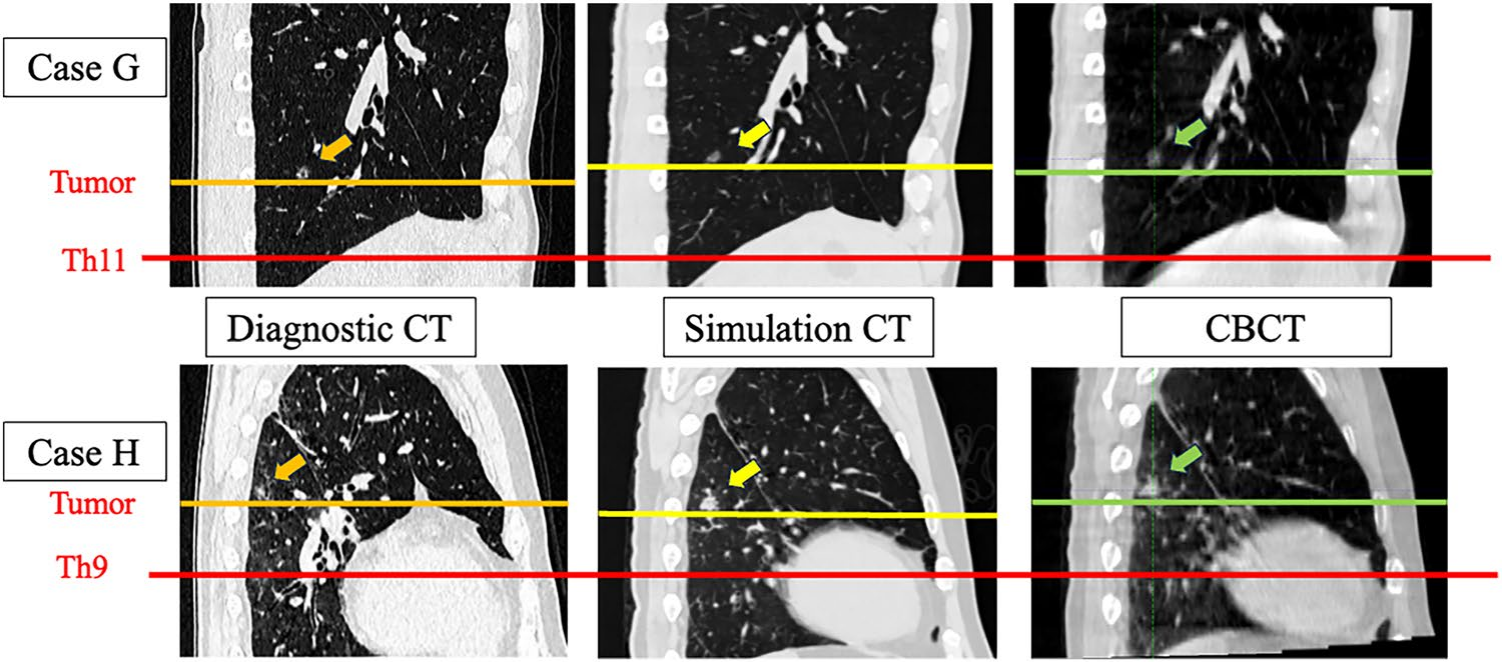
Comparison of tumor positions among diagnostic CT, simulation CT, and CBCT images in the sagittal plane. Red lines denote the positions of the vertebral bodies used as reference points. For patients G and H, Th11 and Th9 served as the reference points. The lower boundary of the tumor is represented by orange (diagnostic CT), yellow (simulation CT), and green (CBCT) lines. *CBCT* cone-beam computed tomography, *CT* computed tomography, *Th* thoracic vertebra

**Fig. 5:**
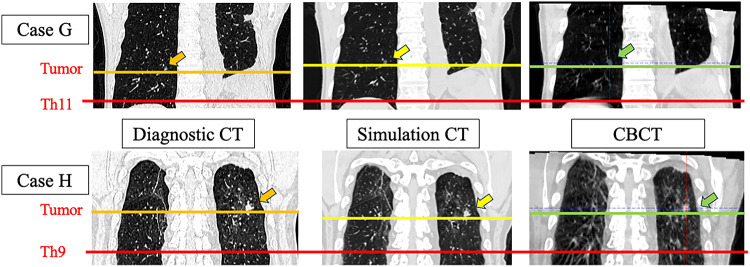
Comparison of tumor positions among diagnostic CT, simulation CT, and CBCT images in the axial plane. Red lines denote the positions of the vertebral bodies used as reference points. For patients G and H, Th11 and Th9 served as the reference points. The lower boundary of the tumor is represented by orange (diagnostic CT), yellow (simulation CT), and green (CBCT) lines. *CBCT* cone-beam computed tomography, *CT* computed tomography, *Th* thoracic vertebra

Figures 4 and 5 display a comparison of tumor positions among the diagnostic CT, simulation CT, and CBCT images on the sagittal and axial planes, respectively. For patient G, the tumor position on diagnostic CT and CBCT was almost the same as that during the deep inspiration breath-hold; nonetheless, on simulation CT, the tumor position was superior to that on diagnostic CT and CBCT. Conversely, for patient H, the tumor position on the diagnostic CT and CBCT was nearly the same as that during the deep inspiration breath-hold; nevertheless, on the simulation CT, it was located inferiorly to those images. This indicates that even with VOXELAN-assisted respiratory management, the patient was breathing shallower or deeper than they did during simulation CT. This may be due to patient anxiety during simulated CT or differences between thoracic and abdominal breathing. Previous studies comparing thoracic-DIBH and abdominal-DIBH have shown that abdominal breath-hold can produce larger cranio-caudal diaphragm excursion and greater lung expansion, often resulting in lower heart and lung doses than thoracic DIBH, although reproducibility may vary by patient [18]. In addition, SGRT-based DIBH stability studies have shown that the external thoracic surface may still move several millimeters depending on breathing pattern, highlighting the clinical relevance of distinguishing thoracic from abdominal breath-holds [19]. Such physiological variations underline the importance of standardized respiratory coaching and patient relaxation during simulation. Even when using VOXELAN for respiratory management, the technologist involved in the simulation CT scan could not detect any differences in the normal respiratory state of the patient. When performing simulated CT, it is essential to ensure that the patient does not experience excessive tension and that differences between thoracic and abdominal breathing do not occur.

Prado et al. evaluated intra-fractional target shift comparisons using two breath-hold systems in lung stereotactic body radiotherapy [20]. They compared spirometry- and surface-guided systems for target detection and reported that the intrafraction shift was reduced when using spirometry-based DIBH rather than surface-guided DIBH, although both methods were equivalent in accuracy for intrafraction control. Additionally, higher superior-inferior shifts were observed in patients with inferior lobe tumors. Their findings demonstrated that the SB system offered slightly better reproducibility, particularly for lower-lobe tumors, which exhibited greater shift. However, both systems maintained an average submillimeter accuracy. Prado et al. concluded that although SGRT is clinically viable, careful consideration is required for lower-lobe cases because of greater diaphragm-related movement. Our findings are consistent with this observation, as the longitudinal direction showed the largest variability even under VOXELAN-guided control. In a similar study, the reproducibility of surface-guided DIBH for lung SBRT was evaluated using a ring-mounted SGRT system with a closed-bore linear accelerator [21]. Seventy-three SGRT-guided abdominal DIBH treatment sessions were conducted, and the tumor and surface positions were assessed using kV-CBCT and SGRT reports. Tumor motion remained within the submillimeter range, and a strong correlation was found between the surface and internal motion in several directions. DIBH significantly reduced the planning target volumes and lung doses. These results, together with our data, demonstrate that SGRT provides clinically acceptable reproducibility for most directions, while residual longitudinal discrepancies require further management.

Fu et al. reported intra-fractional tumor motion in lung stereotactic radiotherapy with DIBH using CBCT images [22]. They analyzed 28 patients who underwent posttreatment CBCT to qualify for tumor shifts, demonstrating that most shifts remained within the applied PTV margins (5 mm vertical/lateral; 8 mm longitudinal), with a slightly larger variation in the longitudinal direction. They mentioned that it was possible to reduce it further in the lateral direction, even though this was not performed. Their reported magnitude of longitudinal variation aligns with the present study, confirming that longitudinal motion remains a key limiting factor for safe margin reduction.

Naumann et al. assessed the feasibility of SGRT for position verification and intra-fraction monitoring during DIBH in SBRT [23]. In 20 patients with lung and liver tumors, SGRT was compared with CBCT and fluoroscopy, exhibiting a high match between the surface and internal positions, with mean deviations of less than 2 mm. The authors concluded that SGRT reliably detected breath-hold reproducibility and intra-fractional motion. Similar to their findings, our study confirmed that VOXELAN effectively maintained positional reproducibility in vertical and lateral directions, reinforcing its clinical reliability.

Sarudis et al. reported surface-guided frameless positioning for lung SBRT [24]. They evaluated CBCT data with free breathing using an SGRT device and compared the dosimetric parameters in radiotherapy planning. According to their report, the intra-fractional shifts of the patients were larger in the longitudinal direction. They stated that monitoring during irradiation with SGRT enabled them to set appropriate margins for the target in the treatment plan as a countermeasure to these problems. The authors concluded that frameless immobilization using SGRT for motion management and respiration monitoring is a feasible approach for lung SBRT. Importantly, our findings corroborate this conclusion. We performed respiratory management on patients when acquiring simulated CT images to ensure the reproducibility of their breathing and referred to the body surface data and respiratory waveforms obtained by VOXELAN installed in the simulation CT room to perform prepositioning in the treatment room. After completing image guidance by matching the tumors with the CBCT images, we performed radiation management during irradiation using VOXELAN in the treatment room. This process considers not only the position of the patient but also the respiratory waveform, contributing to the reproducibility of the position and waveform from the simulation CT to the treatment room on the same coordinate scale using VOXELAN. Furthermore, a European team reported on patterns of practice for respiratory motion management [25], stating that breath-holding is a common technique used in the treatment of lung cancer and concluding that motion-management techniques are important. VOXELAN is based on the light-section method and accurately acquires surface shape. Our findings extend these reports by providing quantitative evidence that integrating surface-based prepositioning and respiratory coaching in a unified workflow improves reproducibility and supports safer margin definition. We conclude that the implementation of motion management and prepositioning using VOXELAN during simulated CT acquisition, as well as body surface monitoring during irradiation, is effective.

The case group examined in this study consisted of cases that were generally similar and eligible for SBRT. Nonetheless, because not all cases were identical, random errors due to differences between cases were included. Additionally, in this analysis, the vertebral structure was assumed fixed and not subjected to respiratory motion when the marker was set. However, in the middle thoracic spine, the vertebrae may have bent slightly during deep breathing compared to the treatment planning phase. Therefore, uncertainty resulting from this effect cannot be ruled out. Furthermore, the number of patients in this study was 35, which may not have been sufficient for statistical analysis. Nevertheless, by ensuring that the sample sizes in each group were similar, statistical errors in the analysis were excluded. Future studies with a larger cohort and integrated intra-fraction monitoring are warranted to validate these findings and to explore potential dosimetric benefits of margin reduction based on SGRT reproducibility.

## Conclusions

We assessed the effects of prepositioning using VOXELAN and respiratory coaching in 35 SBRT cases. The application of VOXELAN for respiratory management and prepositioning enhances the reproducibility of internal target localization. Notably, patients in Group A who adhered to the VOXELAN setup criteria exhibited higher concordance with internal target localization than did those not meeting the criteria. However, even with VOXELAN prepositioning, the tumor position in the longitudinal direction remained inadequately controlled; thus, caution is advised against indiscriminate margin reduction.

## Data Availability

The data that support the findings of this study are not publicly available due to institutional restrictions but are available from the corresponding author upon reasonable request.
